# Elevated Plasma Levels of MT4-MMP and MT6-MMP; A New Observation in Patients with Thyroid Nodules

**DOI:** 10.34172/aim.2023.51

**Published:** 2023-06-01

**Authors:** Khadijeh Shirazkeytabar, S. Adeleh Razavi, Raziyeh Abooshahab, Pouya Salehipour, Mahdi Akbarzadeh, Ahmadreza Soroush, Mehdi Hedayati, Shirzad Nasiri

**Affiliations:** ^1^Department of Surgery, Shariati Hospital, School of Medicine, Tehran University of Medical Sciences, Tehran, Iran; ^2^Department of Surgery, Shariati Hospital, School of Medicine, Tehran University of Medical Sciences, Tehran, Iran; ^3^Department of Research and Development (R&D), Saeed Pathobiology & Genetics Laboratory, Tehran, Iran; ^4^Curtin Medical School, Curtin University, Bentley 6102, Australia; ^5^Department of Medical Genetics, School of Medicine, Tehran University of Medical Sciences, Tehran, Iran

**Keywords:** Glycosyl-phosphatidyl inositol, Matrix metalloproteinases, MT4-MMP, MT6-MMP, Thyroid nodules

## Abstract

**Background:**

Based on the critical role of MT4-MMP and MT6-MMP in carcinogenesis, we focused on MT4-MMP and MT6-MMP circulating levels in patients with thyroid nodules.

**Methods:**

Plasma samples were collected from three groups, including papillary thyroid cancer (PTC; n=30), multinodular goiter (MNG; n=30), and healthy subjects (n=22). Enzyme-linked immunosorbent assay (ELISA) was used to obtain the concentration of MT4-MMP and MT6-MMP in the three groups.

**Results:**

Analysis of data demonstrated increased levels of MT4-MMP (PTC: 4.90±1.35, MNG: 4.89±1.37, and healthy: 3.13±1.42) and MT6-MMP (PTC: 8.29±2.50, MNG: 7.34±2.09, and healthy:5.01±2.13) in thyroid nodules by comparison with healthy subjects (*P*<0.05). There were no significant differences in the levels of the two MT-MMPs between PTC and MNG (*P*>0.05). Increased plasma levels of MT4-MMP (odds ratio=2.48; 95% CI: 1.46–4.19; *P*=0.001) or MT6-MMP (odds ratio=1.81; 95% CI: 1.29–2.53; *P*=0.001) were associated with increased risk of PTC tumorigenesis. Interestingly, a strong positive association was observed between MT4-MMP and MT6-MMP in the three groups (PTC: r=0.766**, *P*=0.000; MNG: r=0.856**, *P*=0.000; healthy r=0.947**, *P*=0.000). Areas under the ROC curve for MT4-MMP and MT6-MMP were 0.82 and 0.96, respectively. At the cutoff value>4.7 (ng/mL), MT4-MMP and MT6-MMP showed a sensitivity of 63.3% and 90.0%, respectively, with 100% specificity.

**Conclusion:**

Our work has led us to imply that the higher levels of MT4-MMP and MT6-MMP are closely linked with both PTC and MNG tumorigenesis. They may probably promote the development of thyroid lesions; however, more research is needed to further clarify the current findings.

## Introduction

 Thyroid cancer is the most prevalent type of malignancy in the head and neck area, with a worldwide increasing and unpredictable incidence.^1^ Papillary thyroid cancer (PTC) is the most prevalent and curable type of thyroid cancer, accounting for 80% of cases.^2^ At present, the diagnosis of PTC is primarily based on fine-needle aspiration biopsy (FNAB) under ultrasound guidance with cytological examination.^3^ However, this method has primary limitations. It is not only an invasive procedure, but 15%-30% of FNAB cytological results are challenging and cannot discriminate between malignant lesions and benign cases; thus, they are given indeterminate reports.^4^ Consequently, lobectomy or thyroidectomy is ultimately an inevitable diagnostic option.^5^ Hence, finding a reliable biomarker to improve the effectiveness of non-invasive diagnostics with high sensitivity and accuracy in these cases^6,7^ is crucial. For this purpose, blood-based biomarkers could appear as a minimally invasive and practical approach to differentiate malignancy from benign cases. In several previous studies, various blood biomarkers have been evaluated extensively. Such biomarkers include vascular endothelial growth factor (VEGF), matrix metalloproteinase 9 (MMP-9), biotinidase, clusterin, cysteine-rich, angiogenic inducer 61 (CYR61), enolase 1, nucleolin and prothymosin alpha (PTMA), Gal-3, and TIMP-1.^8-10^

 Unfortunately, to date, all these blood-based biomarker studies have failed to provide clinical utility due to their lack of sensitivity, specificity, and negative predictive value. Clearly, other blood biomarkers with higher sensitivity and specificity should be identified and validated.

 Cancer initiation and progression are ascribed to the activity of several proteolytic systems. In this regard, the proteolytic activities of matrix metalloproteinases (MMPs) play a significant role in tumorigenesis.^11^ MMPs are one of the most critical and multifunctional extracellular matrix (ECM) proteins belonging to the zinc-dependent proteolytic enzymes.^12^ MMPs play a pivotal role in remodeling ECM associated with tissue alterations.^13^ It has been well known that overexpression of MMPs intensified ECM degradation correlated with angiogenesis, tumor growth, and metastasis.^14,15^ Membrane-type matrix metalloproteinases (MT-MMPs) molecules are an important subfamily within the MMPs family, which are divided into two subgroups: a) transmembrane MT-MMPs (TM-MT-MMPs) and b) glycosylphosphatidylinositol-anchored MT-MMPs (GPI-MT-MMPs).^16^ They participate in cell division, cell surface receptors interruption, and intracellular signals regulation.^16,17^ MT4-MMP or MMP17 and MT6-MMP or MMP25, which belong to group b of the MT-MMPs subfamily, have a unique proteolytic and non-proteolytic function due to their GPI anchorage that holds them in the plasma membrane compared to other MT-MMP members.^18^ MT4-MMP promotes tumor cell proliferation via modulating the epidermal growth factor receptor (EGFR) activation in response to its ligands (TGFα and EGF).^19^ MT6-MMP cleaves galectin-3, a carbohydrate-binding protein, which plays a unique role in inflammation, generating apoptosis and cancer progression.^20^ Several reports in the literature support the association of the upregulation of MT4-MMP and MT6-MMP with the development and progression of human cancers.^21-23^

 Herein, based on the primary function of MMPs in tumor progression and metastasis, we sought to specify the presence of MT4-MMP and MT6-MMP in the circulating system of patients with thyroid nodules, including malignant nodules (PTC) or benign nodules (multinodular goiter; MNG) and explore their potency, together or alone, as diagnostic circulating biomarkers.

## Materials and Methods

###  Patients and Plasma Collection

 This case-control study examined MT4-MMP and MT6-MMP in three groups: PTC, MNG, and healthy subjects. Written informed consent was signed by all participants and provided according to the local ethics committee guideline before the patient enrollment.

 Patients who had undergone hemithyroidectomy or total thyroidectomy in Shariati Hospital, Tehran, Iran, were initially enrolled in the study. None of the patients had received any drug, iodine therapy, or blood transfusion before blood collection. PTC or MNG diagnosis was proven by two pathologists after the surgical removal of tumor tissues. According to the postoperative pathological archives, only patients with tumors clinically defined as PTC but not microPTC (tumor size < 1cm) or MNG were included in the study. Patients with other types of cancer or other disorders (metabolic syndrome, diabetes, liver or kidney dysfunction, etc.) were excluded. Staging for each PTC case was determined using the criteria defined by the 7th edition of the American Joint Committee on Cancer (AJCC) Tumor-Node-Metastasis (TNM) staging system.^24^

 The healthy control groups were individuals who asked for self-rated health. Individuals with good health rates without any thyroid-related disorders (hypo/hyperthyroidism, goiter, autoimmune thyroiditis,^25,26^ and thyroid nodules) were included in the study. To increase the accuracy, four thyroid function test parameters, including TSH, T4, T3, freeT4, and FBS, were evaluated in the healthy control group to confirm health status.

 Five mL venous blood samples were collected from all participants using plasma-collecting tubes containing anticoagulant EDTA. Subsequently, the samples were centrifuged for 10 minutes at 3000 rpm at 4 °C. The obtained plasma was then aliquoted into 1.5-mL Eppendorf microtubes and stored at −80 °C until analysis.

###  Assessment of MT4-MMP and MT6-MMP

 According to the manufacturers’ recommendations, all the collected plasma samples were analyzed by a quantitative sandwich-type ELISA method in three groups (PTC, MNG, and healthy). Concentrations of MT4-MMP and MT-6MMP were measured using human MT4-MMP and MT6-MMP ELISA kits (ZellBio GmbH, Germany.) with a minimum sensitivity of 0.1 ng/mL. The intra-assay percent coefficient of variation (%CV) was 7.1% for MT4-MMP and 6.7% for MT6-MMP. All readings were done on the Tecan Sunrise ELISA microplate reader (Tecan, Austria). All the determinations were done by qualified laboratory staff blinded to clinical data.

###  Statistical Analysis

 A Kolmogorov-Smirnov test was used to check the normality of the plasma levels of MT4-MMP and MT6-MMP in three groups. Statistical comparisons of MT4-MMP and MT6-MMP among the three groups were determined using the one-way ANOVA post hoc tests. The association between the levels of MT4-MMP and MT6-MMP with nominal demographic or clinicopathological parameters was analyzed using independent samples t-test between two states of each parameter. A one-way between-subjects ANCOVA was carried out to ascertain the alterations in the concentrations of MT4-MMP and MT6-MMP in the three groups for the effect of age. Pearson’s correlation coefficient test or Spearman’s test established the correlation between the two GPI-MT-MMPs levels and scale or ordinal demographic or clinicopathological parameters. The logistic regression model evaluated the relationship between the two MT-MMPs plasma levels and clinicopathological characteristics in PTC patients. Receiver operating characteristic (ROC) curve analysis assessed the studied parameters’ efficiency.

 All data analyses were conducted with the IMB SPSS statistics version 20.0 (Chicago, IL, USA) and MedCalc (version 14.8.1, Ostend, Belgium), and graphs were depicted using GraphPad Prism 8.0 (La Jolla, CA, USA) statistical software. A *P* value less than 0.05 was considered statistically significant.

## Results

###  Demographic and Pathological Features

 A total of 82 participants, including 30 PTC patients (22 females and 8 males), 30 patients with MNG (25 females and 5 males), and 22 healthy volunteers (18 females and 4 males), entered our experiment. The mean age of patients with MNG was greater than the PTC and healthy groups (*P* = 0.001). The demographic and clinicopathological features of the study participants are summarized in Table 1.

**Table 1:** Demographic and Pathological Characteristics of the Study Participants

**Parameter**	**PTC**	**MNG**	**Healthy**	***P***** Value **
Patient number	30	30	22	
Gender				
Male	8	5	4	
Female	22	25	18	
Age (Mean ± SD; years)	37.73 ± 12.49	48.10 ± 12.77	38.45 ± 8.24	0.001
Clinical biochemistry tests (Mean ± SD)				
TSH (µIU/mL)			2.34 ± 0.86	
T4 (nmol/L)			106.13 ± 23.42	
T3 (nmol/L)			1.37 ± 0.20	
Free T4 (pmol/L)			15.81 ± 2.01	
FBS (mg/dL)			89.00 ± 5.37	
Tumor size (Mean ± SD; cm)	2.09 ± 1.16			
Histopathology		30		
Multinodular goiter				
Classic PTC	28			
Follicular variant of PTC	2			
Invasion				
Negative	22			
Positive^a^	8			
Lymph node metastasis^b^				
Negative	14			
Positive	16			
TNM stage^c^				
I	22			
II	2			
III	3			
IVA	3			

PTC, papillary thyroid carcinoma; MNG, multinodular goiter. ^a^ Includes extracapsular and/or lymphovascular invasion. ^b^ Includes N1a and/or N1b. ^c^ American Joint Committee on Cancer (AJCC) Tumor-Node-Metastasis (TNM) staging system.

###  MT4-MMP and MT6-MMP Levels in Three Groups

**Figure 1:**
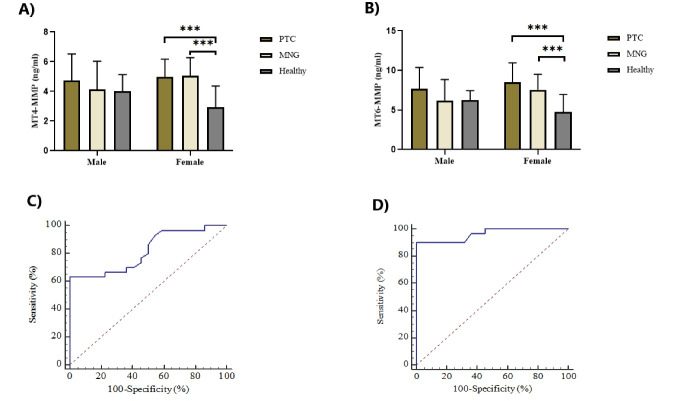
Comparison of MT4-MMP (A) and MT6-MMP (B) Plasma Levels in Male and Female Groups Separately, Using One-Way ANOVA Analysis. Both MT4-MMP and MT6-MMP were significantly elevated in PTC and MNG compared with the healthy subjects in female groups. No significant differences were observed in the plasma levels of two MT-MMPs among males in the three groups (^*^P < 0.001). ROC curves for the ability of MT4-MMP (C) and MT6-MMP (D) to predict PTC patients from healthy subjects. The area under the curves (AUC) for MT4-MMP and MT6-MMP were 0.819 and 0.962, respectively. PTC; papillary thyroid cancer, MNG; multinodular goiter.

 The plasma levels of the two MT-MMPs in PTC patients were significantly higher compared to healthy controls (Table 2), which persisted for both MT-MMPs (ANCOVA, *P* = 0.000) after adjusting for age (ANCOVA, *P* > 0.05) as a covariate. There were no significant differences between patients with PTC and MNG (MT4-MMP: 4.90 ± 1.35 vs. 4.89 ± 1.37 ng/mL, *P* = 1.000; MT6-MMP: 8.29 ± 2.50 vs. 7.34 ± 2.09 ng/mL, *P* = 0.238). Patients with MNG had significantly higher plasma levels of the two MT-MMPs compared to healthy individuals (Table 2). As can be seen in Figures 1A and B, the differences in plasma levels of MT4-MMP and MT6-MMP between women in PTC and MNG groups were statistically significant when compared with healthy subjects (*P* < 0.05). In contrast, the plasma levels of MT4-MMP and MT6-MMP among men did not differ significantly in the three groups (*P* > 0.05).

**Table 2:** MT-MMPs Levels in the Three Groups

	**PTC** **(Mean±SD)**	**MNG** **(Mean±SD)**	**Healthy** **(Mean±SD)**	**ANOVA** ***P*****value**^a^	**Multiple Comparisons** **(Tukey HSD)**	**95% CI**	***P*** **value**^b^
MT4-MMP (ng/mL)	4.90 ± 1.35	4.89 ± 1.37	3.13 ± 1.42	< 0.001	PTC, MNG	(-0.8383, 0.8583)	1.000
					PTC, Healthy	(0.8539, 2.6983)	< 0.001
					MNG, Healthy	(0.8439, 2.6883)	< 0.001
MT6-MMP (ng/mL)	8.29 ± 2.50	7.34 ± 2.09	5.01 ± 2.13	< 0.001	PTC, MNG	(-0.4408, 2.3475)	0.238
					PTC, Healthy	(1.7607, 4.7920)	< 0.001
					MNG, Healthy	(0.8074, 3.8387)	0.001

PTC, papillary thyroid carcinoma; MNG, multinodular goiter; CI, confidence interval. ^a^ *P* values are from the One-Way ANOVA test. A *P* value of < 0.05 was considered statistically significant. ^b^ *P* values are from the Tukey post-hoc test. A *P* value of < 0.05 was considered statistically significant.

###  MT4-MMP and MT6-MMP Levels in Pathological Subgroups of PTC 

 To assess the association of MT4-MMP and MT6-MMP plasma levels with nominal demographic/pathological characteristics of the PTC group, independent samples *t* test was performed to compare parameters between groups (Table 3). Although these MT-MMPs may probably play roles in cancer progression, no significant difference was found between patients with invasion vs. without invasion (MT4-MMP: *P* = 0.419; MT6-MMP: *P*= 0.168) as well as patients with lymph node metastasis vs. without lymph node metastasis (MT4-MMP: *P* = 0.606; MT6-MMP: *P* = 0.216) (Table 3). Table 3 also compares the two MT-MMPs levels between men and women in the MNG and healthy groups.

**Table 3:** Association of MT4-MMP and MT6-MMP Plasma Levels with Nominal Demographic/pathological Characteristics of Studied Groups

**Parameter**		**PTC**		**MNG**		**Healthy**	
		**Mean±SD**	***P***** value**	**Mean±SD**	***P***** value**	**Mean±SD**	***P*****-value**^c^
MT4-MMP	Gender		0.670		0.182		0.181
	Male	4.73 ± 1.79		4.14 ± 1.90		4.00 ± 1.14	
	Female	4.97 ± 1.20		5.04 ± 1.23		2.93 ± 1.43	
	Invasion		0.419				
	Negative	5.06 ± 1.40					
	Positive ^a^	4.64 ± 1.27					
	Lymph node metastasis ^b^		0.606				
	Negative	4.76 ± 1.26					
	Positive	5.03 ± 1.45					
MT6-MMP	Gender		0.426		0.196		0.103
	Male	7.68 ± 2.70		6.22 ± 2.64		6.23 ± 1.23	
	Female	8.51 ± 2.45		7.56 ± 1.95		4.74 ± 2.22	
	Invasion		0.168				
	Negative	8.77 ± 2.54					
	Positive	7.45 ± 2.30					
	Lymph node metastasis		0.216				
	Negative	7.68 ± 2.53					
	Positive	8.83 ± 2.43					

PTC, papillary thyroid carcinoma; MNG, multinodular goiter. ^a^ Includes extracapsular and/, or lymphovascular invasion. ^b^ Includes N1a and/or N1b. ^c^ *P* values are from the independent samples *t*-test. *P* value less than 0.05 was considered statistically significant.

 Furthermore, binary logistic regression analysis revealed that an increased level of plasma MT4-MMP (odds ratio = 2.48; 95% CI: 1.46–4.19; P = 0.001) or MT6-MMP (odds ratio = 1.81; 95% CI: 1.29–2.53; *P* = 0.001) was linked with an elevated risk of PTC tumorigenesis. Binary logistic regression analysis for the two MT-MMPs was also statistically significant for increased risk of MNG (MT4-MMP: odds ratio = 2.40; 95% CI: 1.45–3.99; *P* = 0.001, and MT6-MMP: odds ratio = 1.68; 95% CI: 1.21–2.32; *P* = 0.002).

###  MT4-MMP and MT6-MMP Correlation in Three Groups

Table 4 illustrates a highly significant correlation (PTC: r = 0.766**, *P* = 0.000; MNG: r = 0.856**, *P* = 0.000; healthy r = 0.947**, *P* = 0.000) between MT4-MMP and MT6-MMP plasma levels in the three studied groups. This table also shows that the two MMPs levels had no significant relationship with age, tumor size, and TNM staging.

**Table 4:** Correlation Analysis Between the Two MT-MMPs (MT4-MMP and MT6-MMP) Levels and Scale or Ordinal Demographic/pathological Characteristics

**Parameter**		**PTC**		**MNG**		**Healthy**	
		**r Value**^b^**/ *****P*****Value**^c^	***P***** Value**	**r Value**	***P***** Value**	**r Value**	***P***** Value**
MT4-MMP	Age	0.231	0.220	0.177	0.349	0.304	0.169
	MT6-MMP	0.766^**^	< 0.001	0.856^**^	0.000	0.947^**^	0.000
	Tumor Size	0.157	0.409				
	TNM ^a^	0.005	0.977				
MT6-MMP	Age	0.182	0.336	0.154	0.417	0.233	0.297
	Tumor size	- 0.057	0.764				
	TNM	0.098	0.608				

PTC, papillary thyroid carcinoma; MNG, multinodular goiter ^a^American Joint Committee on Cancer (AJCC) Tumor-Node-Metastasis (TNM) staging system. ^b^ThePearson test has been employed to assess the correlation between MT4-MMP and MT6-MMP levels and quantitative demographic or pathological characteristics. A *P* value of < 0.05 was considered statistically significant. ^c^The Spearman's rho test has been used to assess the relationships between MT4-MMP and MT6-MMP levels and TNM stage. A *P* value of < 0.05 was considered statistically significant. ^**^Correlation is significant at the 0.01 level.

###  ROC Curve Analysis of MT4-MMP and MT6-MMP

 The prediction ability of MT4-MMP and MT6-MMP to distinguish PTC from healthy subjects was evaluated using ROC curve analyses. At the cutoff > 4.7 (mg/mL), MT4-MMP with 63.33% sensitivity and 100% specificity and MT6-MMP with 90.0% sensitivity and 100% specificity had significant prediction power to discriminate PTC from healthy individuals. Areas under the curve (AUC) are illustrated in Figure 1C, D.

## Discussion

 The MT-MMPs are an important subgroup within the MMPs family, with proteolytic and non-proteolytic functions through a transmembrane domain, an amino-terminal link, or a GPI anchor.^16^ The GPI-anchored types of MT-MMPs, including MT4-MMP and MT6-MMP, reside in the lipid raft of the plasma cell membranes and have access to a distinct set of substrates.^18^ Nowadays, increasing evidence suggests that GPI-MT-MMPs may be critical factors in tumorigenesis and involved in cancer progression.^19-21^ In the present study, we investigated the MT4-MMP and MT6-MMP circulating levels in PTC, MNG, and healthy groups and then evaluated the association of their concentration with demographic or clinicopathological characteristics in an Iranian population.

 To our knowledge, these data provided the first evidence of the status of MT4-MMP and MT6-MMP plasma levels in patients with thyroid nodules. The results showed that MT4-MMP and MT6-MMP plasma levels were significantly elevated in the PTC and MNG groups. The existing data suggest that higher plasma levels of MT4-MMP and MT6-MMP could arise by up-regulation of these two proteins in the involved organs, and this may be a factor in both PTC carcinogenesis and MNG pathogenesis. The logistic regression results verified that elevated levels of MT4-MMP or MT6-MMP enhanced the risk of PTC and MNG. Moreover, high levels of MT4-MMP and MT6-MMP were noted in women than men in the three groups. This can be contributed to the small sample size of men. We also considered the effects of age among the three groups, which did not show to be a significant covariate. This experiment also demonstrated the ability of two MT-MMPs as diagnostic biomarkers for PTC. Both MT4-MMP and MT6-MMP plasma levels at the cutoff value of > 4.7 ng/mL had good performance for PTC diagnosis. Further experiments carried out by researchers on multiple tumor tissues or cell lines using reverse transcription-polymerase chain reaction (RT-PCR) and immunostaining procedures concurred with our findings.^27,28^ The expression of MT4-MMP protein was first abundantly found in human breast cancer.^28^ Afterward, the role of MT4-MMP in tumor progression and invasion was discovered in head and neck cancer,^29^ colon cancer,^30^ and gastric cancer.^31^ Identifying MT6-MMP mRNA expression in colon cancer cell lines (SW480),^32^ led to establishing the first investigation of its expression at both mRNA and protein levels in invasive colon cancer.^27^ This study immunohistochemically demonstrated that the expression of MT6-MMP protein was increased in all cases with invasive adenocarcinomas. Moreover, higher expression of MT6-MMP at the mRNA level has been observed in several other human cancers, such as brain tumors,^32^ prostate cancer,^33^ gastric cancer,^31^ and oral tongue squamous cell carcinoma.^22^

 We did not find any significant association between the status of MT4-MMP and MT6-MMP and demographic or disease clinicopathological features. This findingis in contradiction with some previous results reported in the literature. In a study by Cabottaux et al, higher expression of MT4-MMP protein in breast cancer was related to metastatic lymph nodes.^34^ What is surprising is that in their functional study, higher expression of recombinant MT4-MMP in the breast cancer cell line (MDA-MB-231) did not influence cell proliferation or invasion but contributed to tumor growth and progression of lung metastases when inoculated in RAG-1 immunodeficient mice. Ultimately, they suggested that MT4-MMP plays a critical factor in the progression and metastasis of breast cancer. Likewise, MT6-MMP-mediated metastatic dissemination has been noted in colon cancer.^27^ Another study by Wang et al on gastric cancer indicated that the depth of tumor invasion, lymph node metastasis, and widespread serous membrane involvement were significantly linked with higher mRNA and protein expression of MT4-MMP and MT6-MMP.^31^

 The results obtained from our study do not necessarily mean that there is no correlation between the two GPI-MT-MMPs’ expression and adverse pathology parameters. According to the published reports, it seems that MT4- and MT6-MMP require a 3D extracellular tumor microenvironment for migration, invasion, and metastasis rather than 2D plasma conditions. Therefore, while there is no association between the MMPs expression and clinicopathological features in the blood, this association may be present at the tumor level. IHC analysis of thyroid tumors is mandatory for future studies to support this interpretation. The small sample size, especially the sample size of patients with invasion or lymph node metastasis or type III/IV stage, might be another reason for the lack of significant associations between adverse pathology parameters and the status of the two GPI-MT-MMPs. It could also be considered that PTC is intrinsically non-aggressive cancer, and its molecular mechanism is probably not the same as breast, colon, or gastric cancer. The more we investigate, the more we know how PTC progression could relate to GPI-MT-MMPs expression.

**Figure 2:**
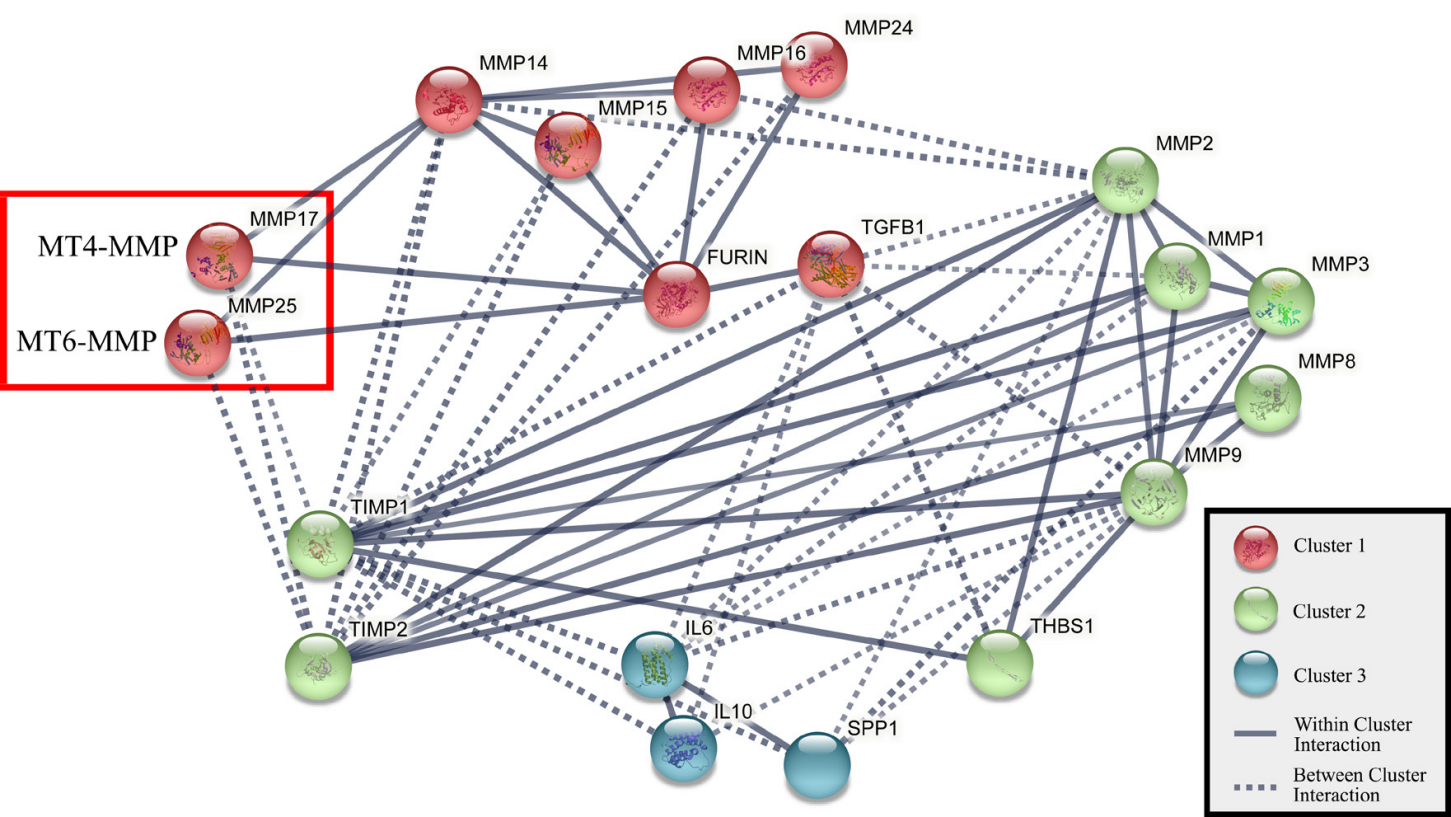
Interaction Pathway of MT-MMP Proteins (MMP14, MMP15, MMP16, MMP17, MMP24, MMP25) Obtained by STRING Protein-Protein Interaction Network (version 11). All interaction scores are above 0.75, and the network consists of 3 clusters: cluster 1 shows MT-MMP proteins, cluster 2 shows soluble MMP proteins, and cluster 3 shows interleukin proteins.

 The marked observation to emerge from the analyses was a strong positive correlation between MT4-MMP and MT6-MMP levels. It suggests that the expression of one molecule may influence the expression of the other. Overall, it may be presumed that the two proteins probably conform to the same transcriptional and/or translational regulation, and their functional role complements each other. Based on these obtained results, we used the STRING protein-protein interaction (PPI) network (version 11) to explore the potential MT4-MMP-MT6-MMP interactions (Figure 2). According to the PPI analysis, MT4-MMP and MT6-MMP are not directly related. Nevertheless, they are connected to furin on one side and MMP14 (MT1-MMP) on the other. Both TM-MT-MMPs and GPI-MT-MMPs consist of an RXR/KR motif at the propeptide domain end that stands for furin recognition site, which cleaves the propeptide and activates the MT-MMP zymogen.^18^ MT1-MMP is recognized as one of the most important members of the MMP family and a key player in cancer progression.^16,18^ Unraveling the exact relationship between the three enzymes, MT1-MMP, MT4-MMP, and MT6-MMP, is essential for more detailed insight into the protein-protein interactions.

## Conclusion

 In conclusion, the results of the present initial study demonstrated higher plasma levels of MT4-MMP and MT6-MMP in both PTC and MNG compared with healthy subjects. In our view, these results suggest the tumorigenesis role of the two markers in thyroid nodules. Since we did not see any significant differences between PTC and MNG, the GPI-MT-MMPs family could not be considered diagnostic blood-based biomarkers to discriminate malignancy from benign cases in thyroid lesions. Nevertheless, research into this issue in terms of indeterminate lesions is already underway.
